# Subantimicrobial Dose Doxycycline Worsens Chronic Arthritis-Induced Bone Microarchitectural Alterations in a Mouse Model: Role of Matrix Metalloproteinases?

**DOI:** 10.3389/fphar.2019.00233

**Published:** 2019-03-20

**Authors:** Ádám Horváth, Bálint Botz, Tamás Kiss, Kata Csekő, Ibolya Kiss, Attila Felinger, Tamara Szabados, Éva Kenyeres, Péter Bencsik, Attila Mócsai, Péter Ferdinandy, Zsuzsanna Helyes

**Affiliations:** ^1^Department of Pharmacology and Pharmacotherapy, Medical School, University of Pécs, Pécs, Hungary; ^2^Molecular Pharmacology Research Group, Centre for Neuroscience, János Szentágothai Research Centre, University of Pécs, Pécs, Hungary; ^3^Department of Radiology, Clinical Centre, University of Pécs, Pécs, Hungary; ^4^Department of Analytical and Environmental Chemistry, Faculty of Sciences, Institute of Chemistry, University of Pécs, Pécs, Hungary; ^5^Environmental Analytical and Geoanalytical Research Group, János Szentágothai Research Centre, University of Pécs, Pécs, Hungary; ^6^Department of Pharmacology and Pharmacotherapy, Faculty of Medicine, University of Szeged, Szeged, Hungary; ^7^Department of Biochemistry, Faculty of Medicine, University of Szeged, Szeged, Hungary; ^8^Pharmahungary Group, Szeged, Hungary; ^9^Department of Physiology, Faculty of Medicine, MTA-SE “Lendület” Inflammation Physiology Research Group of the Hungarian Academy of Sciences, Semmelweis University, Budapest, Hungary; ^10^Department of Pharmacology and Pharmacotherapy, Faculty of Medicine, Semmelweis University, Budapest, Hungary; ^11^Chronic Pain Research Group, National Brain Research Program, Medical School, University of Pécs, Pécs, Hungary; ^12^PharmInVivo Ltd., Pécs, Hungary

**Keywords:** rheumatoid arthritis, matrix metalloproteinases, K/BxN serum-transfer arthritis, subantimicrobial dose doxycycline, bone homeostasis, *in vivo* optical imaging, micro-CT, gelatin zymography

## Abstract

**Background:** Rheumatoid arthritis (RA) is a chronic inflammatory joint disease hallmarked by irreversible damage of cartilage and bone. Matrix metalloproteinases (MMPs) involved in connective tissue remodeling play an important role in this process. Numerous MMPs have been examined in humans and animals, but their functions are still not fully understood. Therefore, we investigated the role of MMPs in the K/BxN serum-transfer model of RA with the broad-spectrum MMP inhibitor subantimicrobial dose doxycycline (SDD) using complex *in vivo* and *in vitro* methodolgy.

**Methods:** Chronic arthritis was induced by repetitive i.p. injections of K/BxN serum in C57BL/6J mice. SDD was administered daily in acidified drinking water (0.5 mg/mL, 80 mg/kg) during the 30 days experimental period. Mechanonociceptive threshold of the paw was evaluated by aesthesiometry, grasping ability by grid test, arthritis severity by scoring, neutrophil myeloperoxidase activity by luminescence, vascular hyperpermeability and MMP activity by fluorescence *in vivo* imaging and the latter also by gelatin zymography, bone structure by micro-computed tomography (micro-CT). Plasma concentrations of doxycycline were determined by liquid chromatography-mass spectrometry analysis.

**Results:** K/BxN serum induced significant inflammatory signs, mechanical hyperalgesia, joint function impairment, increased myeloperoxidase activity and vascular hyperpermeability. Significant increase of MMP activity was also observed both *in vivo* and *ex vivo* with elevation of the 57–60, 75, and 92 kDa gelatinolytic isoforms in the arthritic ankle joints, but neither MMP activity nor any above described functional parameters were influenced by SDD. Most importantly, SDD significantly reduced bone mineral density in the distal tibia and enhanced the Euler number in the ankle. Arthritis-induced microarchitectural alterations demonstrating increased irregularity and cancellous bone remodeling, such as increased Euler number was significantly elevated by SDD in both regions.

**Conclusion:** We showed increase of various MMP activities in the joints by *in vivo* fluorescence imaging together with *ex vivo* zymography, and investigated their functional significance using the broad-spectrum MMP inhibitor SDD in the translational RA model. This is the first demonstration that SDD worsens arthritis-induced bone microarchitectural alterations, but it appears to be independent of MMP inhibition.

## Introduction

Rheumatoid arthritis (RA) is a progressive, chronic inflammatory joint disease leading to irreversible articular cartilage and bone destruction. It is one of the most common musculoskeletal disorder causing physical disability with a worldwide prevalence of approximately 1% (Gibofsky, 2012). Despite the therapeutic revolution in the last decades, the treatment of RA is not fully resolved. Although the novel biologics can significantly reduce synovitis and structural progression, they are far from being ideal drugs due to their high costs, ineffectiveness for chronic pain and sometimes serious side effects resulting from immunosuppression (Smolen et al., 2016; McWilliams and Walsh, 2017). Therefore, further research is needed to precisely explore its pathophysiological mechanisms, identify crucial mediators, and find new potential drug targets. These may include matrix metalloproteinases (MMPs), which are important players of joint damage in arthritic conditions, most importantly in RA (Rose and Kooyman, 2016).

MMPs are secreted or membrane-bound enzymes involved in the family of calcium- and zinc-dependent endopeptidases. Their major function is degrading the extracellular matrix, but they are also capable of cleaving certain non-matrix peptides (e.g., cytokines, chemokines, growth factors, cell surface receptors etc.) (Van Lint and Libert, 2007; Fingleton, 2017). They have crucial roles in physiological regulation of embryonic development, tissue remodeling and woundhealing. Furthermore, they are involved in several pathophysiological processes, mainly in “collagenolytic” diseases associated with connective tissue destruction (e.g., arthritic diseases, cancer, atherosclerosis, pulmonary emphysema, chronic inflammatory skin diseases etc.) (Tokito and Jougasaki, 2016;Amar et al., 2017).

The most investigated MMPs in RA are collagenases (MMP-1, MMP-8 and MMP-13), gelatinases (MMP-2 and MMP-9), MMP-3 from stromelysins and MMP-14 from membrane-type (MT) MMPs (Rose and Kooyman, 2016). MMP-1 (interstitial collagenase or human fibroblast collagenase) is a ubiquitously expressed collagenase, which is the earliest MMP excessively produced under several pathological circumstances (Burrage, 2006; Shi et al., 2012). In RA it is originated from the synovium and the cartilage, and together with MMP-3 (stromelysin-1)they have been considered to be useful biomarkers for early diagnosis, disease activity and therapeutic efficacy (Green et al., 2003; Fiedorczyk et al., 2006). MMP-8 (collagenase-2 or neutrophil collagenase) is produced mainly by neutrophils, but is also expressed in chondrocytes and synovial fibroblasts. Surprisingly, its role is clearly protective in arthritic tissues confirmed by studies using MMP-8-deficient mice (Cox et al., 2010; Garcia et al., 2010). MMP-13 (collagenase-3) is expressed predominantly in the chondrocytes and cleaves most efficiently the type II collagen, which is the main matrix component of the articular cartilage. Therefore, it is not surprising to be a potential therapeutic target in RA and osteoarthritis (OA) (Singh et al., 2013). Expression of MMP-2 (gelatinase A) and MMP-9 (gelatinase B) are also elevated in arthritis (Dreier et al., 2004; Duerr et al., 2004), but interestingly they have distinct roles in RA. MMP-2 knockout mice showed significantly increased, while MMP-9-deficient ones significantly reduced severity of arthritis in comparison with their wildtypes, suggesting a protective and a deleterious role of these enzymes in collagen antibody-induced arthritis, respectively (Itoh et al., 2002). Among the MT-MMPs, MMP-14 (MT1-MMP) plays predominant role in joint disorders. It is overexpressed in arthritic cartilage, fibroblasts and osteoclasts activating also proMMP-2 and proMMP-13, mediating bone resorption and promoting proinflammatory gene expression in macrophages (Burrage, 2006; Rose and Kooyman, 2016; Fingleton, 2017).

Although the role of the abovementioned MMPs has already been examined in several experimental arthritis models, surprisingly there are only two studies focusing on MMPs (MMP-8 and -13) in the K/BxN serum-transfer murine arthritis, which is one of the most translational RA model (Garcia et al., 2010; Singh et al., 2013). This is a commonly used inducible model of RA, in which transient polyarthritis is evoked in healthy recipients by passive transfer of arthritogenic serum originating from a spontaneously arthritic transgenic mouse strain. The serum contains primarily anti-glucose-6-phosphate isomerase antibodies, which form immune complexes and trigger RA-like joint inflammation and destruction (Korganow et al., 1999). The main advantages of this model are that it is suitable for studying the B- and T-cell independent immunological mechanisms and in case of repeated serum injection the RA-associated chronic pain with neuropathic components (Korganow et al., 1999; Christianson et al., 2010). Although the pharmacological interventions with MMP inhibitors are also valuable tools to examine the roles of MMPs, in this model their functions had only been studied using specific knockout mice.

Therefore, we investigated the activity and roles of MMPs in the K/BxN serum-transfer arthritis model with the non-selective MMP inhibitor subantimicrobial dose doxycycline (SDD) using a complex *in vivo* and *in vitro* methodolgy. In the present study we showed the increase of various MMP activities in the joints by *in vivo* fluorescence imaging together with *ex vivo* zymography, as well as demonstrated for the first time that SDD worsens arthritis-induced bone microarchitectural alterations most probably independently of MMP inhibition.

## Materials and Methods

### Animals

Experiments were carried out on 12–20 weeks old male C57BL/6J mice weighing 20–30 g. They were bred and kept in the Laboratory Animal House of the Department of Pharmacology and Pharmacotherapy, University of Pécs in 325 × 170 × 140 mm sized cages under a 12 h light/dark cycle at 24–25 °C, provided with standard mouse chow and water *ad libitum*. The total number of animals used in the experiments were 33 (17 in the 30 days and 16 in the 16 days experimental series).

### Ethics Statement

All experiments were performed according to European legislation (Directive 2010/63/EU) and Hungarian Government regulation (40/2013., II. 14.) on the protection of animals used for scientific purposes, complied with the recommendations of the International Association for the Study of Pain. The studies were approved by the Ethics Committee on Animal Research of University of Pécs (license No.: BA 02/2000–2/2012).

### Induction of the Arthritis

Arthritis was induced by repeated intraperitoneal (i.p.) injection of 150 μL of arthritogenic K/BxN serum on the days 0, 3, 10, and 20. Repeated administration was applied to evoke more persistent, long-lasting arthritis. K/BxN sera were obtained from the spontaneously arthritic transgenic K/BxN mice bred and kept in the Animal House of the Department of Physiology, Semmelweis University, Budapest, Hungary. Control animals received non-arthritogenic BxN serum from healthy BxN littermates of K/BxN animals following the same protocols (Borbély et al., 2015).

### Preparation of Doxycycline-Treated Drinking Water

Doxycycline was administered in subantimicrobial dose (below 100 mg/kg), therefore 100 mg doxycycline hyclate (Sigma-Aldrich, St. Louis, MO, United States) was dissolved in 200 mL acidified water (0.5 mg/mL). Since there are no data for chronic doxycycline use in arthritis, the dosage and concentration were based on earlier publications related to other issues (Prall et al., 2002; Marx et al., 2014). The estimated daily consumption of an adult mouse was 5 mL resulting in an approximately 80–85 mg/kg oral dose. To avoid the precipitate formation and reach the expected doxycycline plasma concentration, the water was acidified to pH of 3.2 with 37% hydrochloric acid (Merck, Darmstadt, Germany). The pH of the drinking water was measured by Radelkis Laboratory Digital OP-211 pH meter (Radelkis Ltd., Budapest, Hungary). Mice provided with acidified tap water were used for controls. The water was maintained in standard, clear mouse water bottles (250 mL; Acéllabor Ltd., Vecsés, Hungary) placed in complete mouse cage setups and covered with aluminum foil to prevent the photolysis of doxycycline. All water bottles were changed every other day by the research staff.

### Experimental Design

**FIGURE 1:**
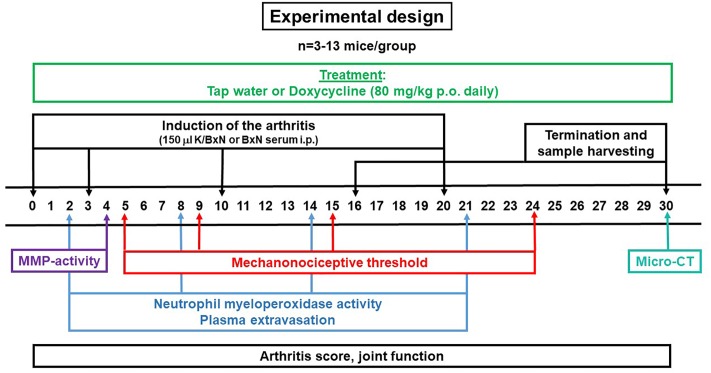
Schematic drawing describing the experimental design of K/BxN serum-transfer arthritis model.

The study was performed in two series as outlined in Figure 1. Daily doxycycline treatment (approximately 80 mg/kg, p.o.) was started on day 0 and continued until day 30 in the first and until day 16 in the second series. The mechanonociceptive threshold of the hind paws were evaluated on days 5, 9, 15, and 24, neutrophil myeloperoxidase (MPO) activity and plasma extravasation on days 2, 8, 14, and 21, MMP activity on day 4, changes of the periarticular bone structure on day 30, arthritis severity and joint function every day during the 30 days experimental period. Following the micro-computed tomography (micro-CT) analysis, animals were euthanized, blood samples were collected for plasma concentration analyis of doxycycline and ankle joints were removed for gelatin zymography. Water consumption was checked every other day, while body weight was measured every day.

### Evaluation of Disease Severity

Visible arthritic signs (hind limb edema and hyperemia) were semiquantitatively scored using a scale of 0–10 (0–1.5: healthy, 1.5–2.5: minimal signs referred to disease, 2.5–4: mild inflammation, 4–7: moderate inflammation, 7–10: severe inflammation) (Jakus et al., 2009; Horvath et al., 2017).

### Evaluation of Mechanonociception

The dynamic plantar aesthesiometer (Ugo Basile 37400, Comerio, Italy) was used for the assessment of the mechanosensitivity of plantar surface of the hind paw. Mice were placed into acrylic glass boxes with wire grid floor, then after acclimation the plantar surface was touched with a straight metal filament lifting with increasing upward force (maximum force of 10 g with 4 s latency) until the animal withdrew his paw. Mechanical hyperalgesia was represented as a percentage decrease of the initial (before serum injection) withdrawal thresholds (Horvath et al., 2016).

### Evaluation of Joint Function

The grasping ability correlating with joint function was determined using the grid test in the K/BxN serum-transfer arthritis model. Mice were placed on a horizontal wire grid, then it was turned over and the latency to fall was determined. The grid was maintained in horizontal position for a maximum of 20 s (Jakus et al., 2009).

### *In vivo* Bioluminescence Imaging of Neutrophil MPO Activity

Neutrophil MPO-derived reactive oxygen species (ROS) production and the enzyme activity were assessed with luminol-derived bioluminescence. Luminol (5-amino-2,3-dihydro-1,4-phthalazine-dione) sodium salt (150 mg/kg, Gold Biotechnology, Olivette, MO, United States) dissolved in sterile phosphate buffered saline (PBS, 30 mg/mL) was injected i.p. into anesthetized mice. They were anesthetized using ketamine (120 mg/kg i.p.; Calypsol, Gedeon Richter Plc., Budapest, Hungary) and xylazine (6 mg/kg i.p.; Sedaxylan, Eurovet Animal Health B.V., Bladel, Netherlands). Bioluminescence imaging was performed 10 min post-injection using the IVIS Lumina II (PerkinElmer, Waltham, MA, United States; 120 s acquisition, Binning = 8, F/Stop = 1). Identical Region of Interests (ROIs) were applied around the ankles and luminescence was expressed as total radiance (total photon flux/s) (Botz et al., 2014).

### *In vivo* Fluorescence Imaging of Plasma Extravasation

Plasma extravasation was visualized by IR-676-based fluorescence imaging. IR-676 vascular fluorescent dye (0.5 mg/kg, Spectrum-Info Ltd., Kyiv, Ukraine) dissolved in 5% (v/v) aqueous solution of Kolliphor HS 15 (polyethylene-glycol-15-hydroxystearate; Sigma-Aldrich, St. Louis, MO, United States) was injected intravenously (i.v.) into anesthetized mice (120/6 mg/kg ketamine-xylazine i.p.). Fluorescence imaging was performed 20 min post-injection using the IVIS Lumina II (PerkinElmer, Waltham, MA, United States; auto acquisition time, Binning = 8, F/stop = 2, excitation/emission filter: 640/700 nm). Data were analyzed and ROIs were drawn around the ankle joints. Fluorescence was expressed as radiant efficiency ([photons/s/cm^2^/sr]/[μW/cm^2^]) (Botz et al., 2015).

### *In vivo* Fluorescence Imaging of MMP Activity

MMP activity was assessed *in vivo* using MMPSense 750 FAST (PerkinElmer, Waltham, MA, United States), an activatable fluorescent imaging agent for MMP-2, -3, -7, -9, -12, and -13 according to the manufacturer’s instructions (2 nmol/subject i.v.). Measurements were performed with IVIS Lumina II (PerkinElmer, Waltham, MA, United States; auto acquisition time, Binning = 2, F/stop = 1, excitation/emission filter: 745/800 nm) 6 h later. ROIs were applied around the ankles and fluorescence was expressed as radiant efficiency ([photons/s/cm^2^/sr]/[μW/cm^2^]) (Borbély et al., 2015).

### *In vivo* Micro-CT Analysis of the Region of Ankle Joints

Micro-CT imaging was performed in a self-control manner before and 30 days after the induction of arthritis. The quantitative values calculated from the pictures at the end of the study were compared to the initial images of the same mice before the experiment. The right ankle joints were scanned using a 17.5 μm voxel size by a SkyScan 1176 *in vivo* micro-CT (Bruker, Kontich, Belgium). After reconstruction of the scans the bone structural changes were evaluated using the CT Analyser^®^ software. Standardized ROIs were drawn around the periarticular region of the distal tibia and fibula, as well as the ankle including both the tibio-tarsal and tarso-metatarsal joints. In these ROIs bone mineral density, bone surface density, number of pores, volume of open pores, percent increase of open pore volume, and the Euler number, a measure of trabecular connectedness were evaluated (Borbély et al., 2015).

### Detection of MMP Activities by Gelatin Zymography

At the end of both series of the experiment, on days 16 and 30, mice were euthanized with sodium pentobarbital (100 mg/kg i.p.; Euthanimal, Alfasan Nederland B.V., Woerden, Netherlands) and ankle joints were removed to assess MMP activities. First the ankle joints were homogenized in a 4 times volume of homogenization buffer containing 50 mM Tris base (Merck, Darmstadt, Germany) and 1 mL 0.5% Triton (Sigma-Aldrich, St. Louis, MO, United States) dissolved in 500 mL distilled water for 2 × 10 s at 20,000 rpm with T25 digital ULTRA-TURRAX homogenizer (IKA-Werke GmbH&Co. KG, Staufen, Germany). Than the joint homogenates were centrifuged at 4°C for 10 min at 10,000 rpm and the supernatants were collected for gelatin zymography. Gelatinolytic activities of MMPs were examined as previously described (Bencsik et al., 2014, 2015). Briefly, 8% polyacrylamide gels were copolymerized with gelatin (2 mg/mL, type A from porcine skin, Sigma-Aldrich, St. Louis, MO, United States), and 25 μg of protein per lane was loaded. An internal standard (American Type Culture Collection, Manassas, VA, United States) was loaded into each gel to normalize activities between gels. After electrophoresis (90 V, 90 min), gels were washed with zymogram renaturation buffer (Novex, Carlsbad, CA, United States) for 40 min. Samples were incubated for 20 h at 37°C in zymogram development buffer (Novex, Carlsbad, CA, United States).

In a separate set of experiments, one sample from non-arthritic and arthritic doxycycline-free experimental groups was loaded into the gel in 4 replicates. After renaturation, the gel was cut into 4 pieces, which were separately incubated in development buffer containing doxycycline hyclate at 0, 0.05, 0.1, or 0.2 μg/mL concentrations, respectively, in accordance with the plasma levels of the doxycycline-treated animals. In another setup 3 pieces of the gel were separately incubated with 2, 20, or 200 μg/mL doxycycline hyclate in comparison with the previously applied 0 and 0.2 μg/mL concentrations to reveal whether these higher concentrations above the originally measured plasma levels are able to inhibit MMP activity.

Gels were then stained with 0.05% Coomassie brilliant blue (Sigma-Aldrich, St. Louis, MO, United States) in a mixture of methanol-acetic acid-water [2.5:1:6.5 (v/v)] and destained in aqueous 4% methanol-8% acetic acid (v/v). Gelatinolytic activities were detected as transparent bands against the dark-blue background. Gels were scanned in a transilluminator and band intensities were quantified by Quantity One software (BioRad, Hercules, CA, United States), and expressed as the ratio to the internal standard, and presented in arbitrary units. For positive controls, gelatinase zymography standard containing human MMP-2 and -9 (Chemicon Europe Ltd., Southampton, United Kingdom) was used. For negative control, lanes containing tissue samples were cut off after renaturation and were separately incubated for 20 h at 37°C in development buffer in the presence of the calcium chelator EGTA [ethylene glycol-bis(2-aminoethylether)-N,N,N′,N′-tetraacetic acid; 10 mM]. Since no gelatinolytic activities could be seen at all, we concluded that the all visible bands derive from MMP activities (data not shown).

### Measurement of Water Consumption and Plasma Concentration of Doxycycline

Mice were kept with a maximum of 8 mice/cage density. Water consumption was measured every other day by weighing the water bottles and measuring the volume of the residual water for each cage until day 30. At the end of both series of the experiment, on days 16 and 30, animals were euthanized with sodium pentobarbital (100 mg/kg i.p.) and blood was taken by cardiac puncture to analyze the plasma concentrations of doxycycline.

The plasma concentrations were determined by liquid chromatography-mass spectrometry (LC-MS) system (Ruz et al., 2004). Stock solutions of doxycycline hyclate, oxytetracycline hydrochloride (≥95%; Sigma-Aldrich, St. Louis, MO, United States) and calibration standards were prepared by weighing 10 mg of the reference standards. These were transferred to individual 10 mL volumetric flasks, diluted with solvent mixture (1% (v/v) acetic acid in methanol:water = 20:80) to obtain a concentration of 1 mg/mL, and stored at 2–8°C protected from light. Solutions of 5000, 2500, 250, 25, 2.5, 1.25, and 0.625 ng/mL concentrations served as calibration standards.

Plasma samples (300 μL) in Eppendorf tubes (2 mL) were measured by spiking with 2 μL of 50 ng/mL oxytetracycline internal standard solution. After mixing, 20 μL of 1 mol/L trichloroacetic acid (Fluka, Buchs, Switzerland) was added, vortex-mixed for 1 min and centrifuged for 15 min at 14 500 rpm. The supernatants were transferred to autosampler vials and 20 μL was injected into the LC–MS–MS system (Agilent LC-MSD-TRAP-XCT_plus, Santa Clara, CA, United States). Ionization parameters and ion optics voltages were optimized for the detection of the oxytetracycline and doxycycline standards (250 ng/L). The Agilent ChemStation and Agilent LC/MSD Trap softwares were applied. Linearity, precision, accuracy, specificity and stability were validated, all results were within the acceptable range.

### Measurement of Phospholipase A2 Activity and Prostaglandin E2 Level in the Joint Homogenates

Cytosolic phospholipase A2 (cPLA2) activity was measured by the colorimetric cPLA2 assay kit (Abcam, Cambridge, United Kingdom; ab133090; sensitivity: 3.5–42 nmol/min/mL) from the tibio-tarsal joint homogenates according to the manufacturer’s protocol. Based on the results of pilot experiments, our undiluted samples were measured in duplicates. The absorbance was recorded at 410 nm using a plate reader (Fluostar Optima, BMG Labtech, Ironmass Consulting Ltd., Budapest, Hungary) and the enzyme activity values were calculated as nmol/min/mL.

Prostaglandin E2 (PGE2) levels from the same homogenates were measured by a colorimetric ELISA kit (antibodies-online GmbH, Aachen, Germany; ABIN365349; detection range: 0.4–80 pg/mL, sensitivity: 0.2 pg/mL) according to the recommendations of the manufacturer in duplicates at 450 nm using on the same plate reader. PGE2 concentration values were calculated as pg/mL by a double logarithmic depiction of the standard curve followed by regression analysis and fitting the samples’ absorbance values to the best fit linear regression.

### Statistical Analysis

Statistical analysis was performed using GraphPad Prism. Results were expressed as means ± standard errors of means (S.E.M.). Arthritis severity, mechanical hyperalgesia, joint function, water consumption, change of body weight and *ex vivo* MMP activity were evaluated by two-way analysis of variance (ANOVA) followed by Bonferroni’s multiple comparison test, *in vivo* luminescence and fluorescence imaging, *in vitro* MMP activity, PLA2 activity and PGE2 level measurements by one-way ANOVA followed by Bonferroni’s multiple comparison test, micro-CT data by two-way ANOVA followed by Sidak’s multiple comparison test. In all cases *p* < 0.05 was considered to be statistically significant.

## Results

### Doxycycline Does Not Decrease Either *in vivo* or *ex vivo* MMP Activity in the Arthritic Ankle Joints

**FIGURE 2:**
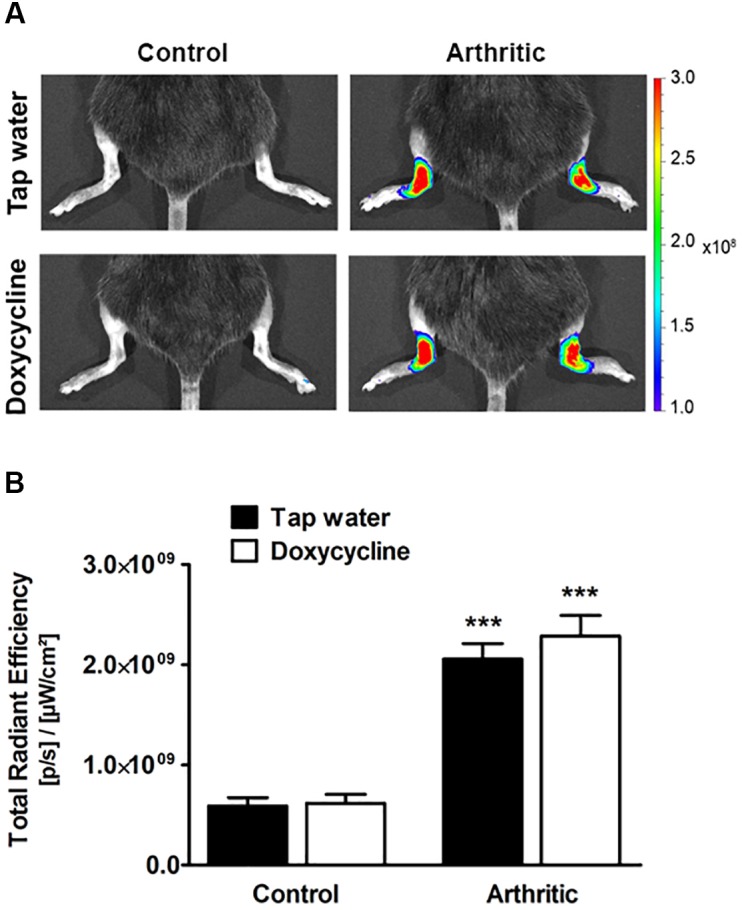
Doxycycline does not inhibit arthritis-induced *in vivo* MMP activity in the ankle joints. **(A)** Representative fluorescence images illustrating *in vivo* MMP activity and **(B)** its quantitative analysis in the ankle joints of non-arthritic (*n* = 3) and arthritic (*n* = 8) mice treated with doxycycline (80 mg/kg p.o. every day during the 30 days experimental period) as compared to the non-arthritic (*n* = 3) and arthritic (*n* = 8) tap water-consumed controls 4 days after arthritis induction. Data are shown as means ± S.E.M. of *n* = 3–8 mice/group, ^∗∗∗^*p* < 0.001 vs. respective non-arthritic controls (one-way ANOVA followed by Bonferroni’s multiple comparison test).

**FIGURE 3:**
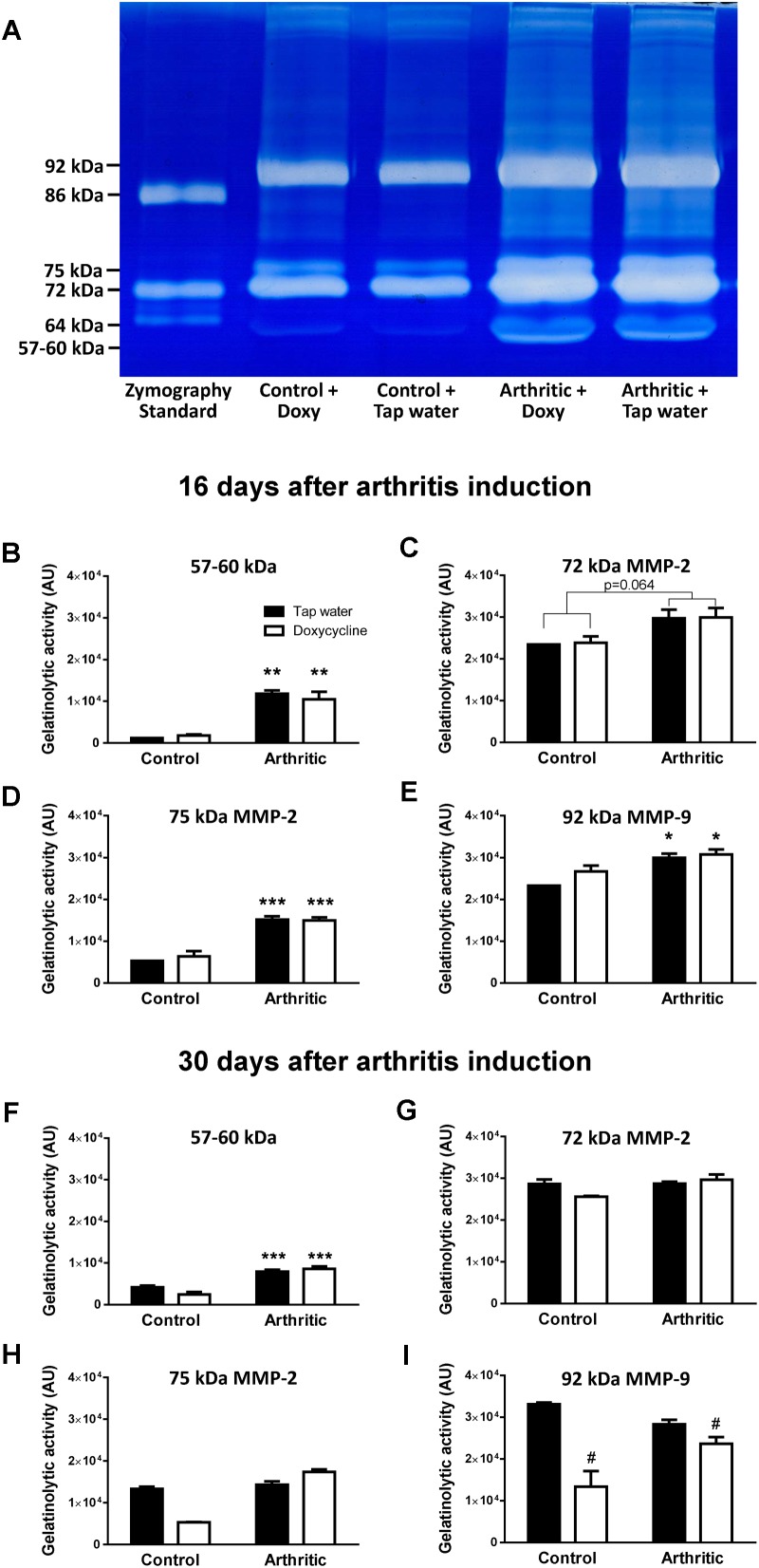
Doxycycline does not inhibit arthritis-induced *ex vivo* MMP activity in the ankle joints. **(A)** Representative gel image illustrating *ex vivo* MMP activity from homogenized mouse ankle joints 16 days after arthritis induction. **(B–E)** Quantitative analysis of MMP activity in the ankle joints of non-arthritic control and arthritic mice treated with doxycycline (80 mg/kg p.o. every day during the 16 days experimental period) compared to the non-arthritic control and arthritic controls consuming tap water 16 days after arthritis induction. **(F–I)** Quantitative analysis of *ex vivo* MMP activity in the ankle joints of non-arthritic control and arthritic mice treated with doxycycline (80 mg/kg p.o. every day during the 30 days experimental period) compared to the non-arthritic control and arthritic controls consuming tap water 30 days after arthritis induction. Data are shown as means ± S.E.M of *n* = 2–5 mice/group, ^∗^*p* < 0.01, ^∗∗^*p* < 0.001, ^∗∗∗^*p* < 0.0001 vs. respective non-arthritic controls; ^#^*p* < 0.01 vs. respective tap water consuming mice (two-way ANOVA followed by Bonferroni’s multiple comparison test).

Arthritis induced a significant increase of fluorescent signal indicating *in vivo* MMP activity in the ankle joints on day 4, but significant difference did not occur between the tap water- and SDD-treated arthritic groups (Figure 2A,B). Sixteen days after arthritis induction 57–60 kDa MMP isoform, 75 kDa MMP-2 and 92 kDa MMP-9, but at 30 days only 57–60 kDa isoform increased significantly in the ankle joint homogenates of both arthritic groups as compared to the non-arthritic controls (Figure 3A–I). On day 30, interestingly, remarkably elevated activity of 92 kDa MMP-9 was observed not only in the tap water-consumed arthritic, but also in the non-arthritic group, which was significantly reduced by SDD in both groups (Figure 3I). Activity of 72 kDa isoform was similar in both non-arthritic and arthritic groups on both days 16 and 30 (Figure 3C,G).

### Doxycycline Does Not Influence Arthritis-Induced Clinical Signs, Mechanical Hyperalgesia, and Joint Function Impairment

**FIGURE 4:**
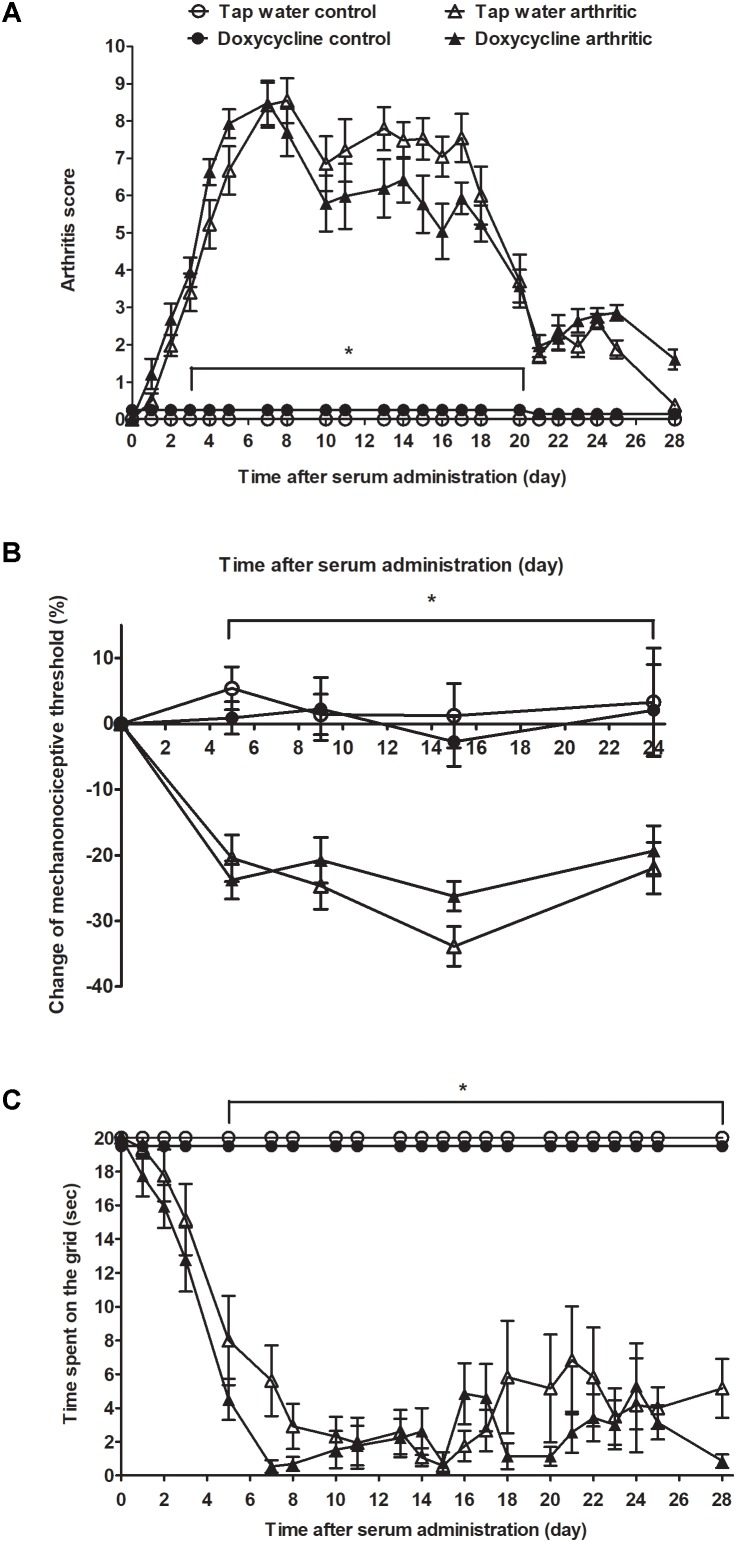
Doxycycline has no effect on K/BxN serum-induced joint inflammation, mechanical hyperalgesia and grasping ability deterioration. Alterations of the **(A)** semiquantitative clinical score, **(B)** mechanonociceptive threshold, and **(C)** time spent on the grid in arthritic (*n* = 13) and non-arthritic (*n* = 5) mice treated with doxycycline (80 mg/kg p.o. every day during the 30 days experimental period) as compared to tap water consuming arthritic (*n* = 12) and non-arthritic animals (*n* = 3). Data are shown as means ± S.E.M. of *n* = 3–13 mice/group, ^∗^*p* < 0.05 vs. respective non-arthritic controls (two-way ANOVA followed by Bonferroni’s multiple comparison test).

Considerable paw edema and hyperemia developed few days after serum injection in the arthritic groups, which reached its maximum on day 7. Then the severity of arthritis decreased slightly by the 2nd boost injection (day 10), which stabilized the disease symptoms. After day 17 the clinical score decreased steeply and despite the 3^rd^ boost injection inflammation did not increase remarkably (Figure 4A). Mechanical hyperalgesia (tap water consuming arthritic group: from 8.81 ± 0.1 to 6.99 ± 0.36 g, doxycycline-treated arthritic group: from 8.93 ± 0.09 to 6.79 ± 0.25 g) and the time spent on the grid were significantly reduced 5 days after arthritis induction and remained unchanged during the 30 days experimental period (Figure 4B,C). SDD treatment did not influence any of the parameters, the severity of clinical signs, the mechanical hyperalgesia and the joint function impairment were similar to the tap water consuming arthritic mice (Figure 4A–C).

### Doxycycline Does Not Influence the Neutrophil MPO Activity and Plasma Extravasation in the Arthritic Ankle Joints

**FIGURE 5:**
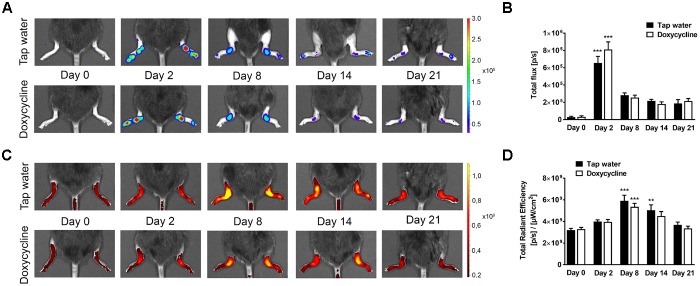
Doxycycline does not alter arthritis-induced neutrophil MPO activity and plasma extravasation. **(A)** Representative bioluminescence and **(B)** fluorescence images illustrating MPO activity and plasma extravasation, respectively, and **(C,D)** their quantitative analysis in the ankle joints of arthritic mice treated with doxycycline (80 mg/kg p.o. every day during the 30 days experimental period; *n* = 13) as compared to the tap water consuming controls (*n* = 12) on days 0, 2, 8, 14, and 21. Data are shown as means ± S.E.M. of *n* = 12–13 mice/group, ^∗∗^*p* < 0.001, ^∗∗∗^*p* < 0.001 vs. respective day 0 controls (one-way ANOVA followed by Bonferroni’s multiple comparison test).

Both arthritic groups showed intensive luminol-derived bioluminescence signal in the ankle joints reaching the maximum on day 2, but SDD had no inhibitory effect at any of the time points (Figure 5A,B). Plasma extravasation was similarly high in the arthritic ankle joints of both groups in the early phase, which increased further until day 8, then decreased slightly until day 21. However, significant difference was also not observed between the groups (Figure 5C,D).

### Doxycycline Significantly Increases Trabecular Connectivity and Reduces Bone Mineral Density in the Periarticular Region

**FIGURE 6:**
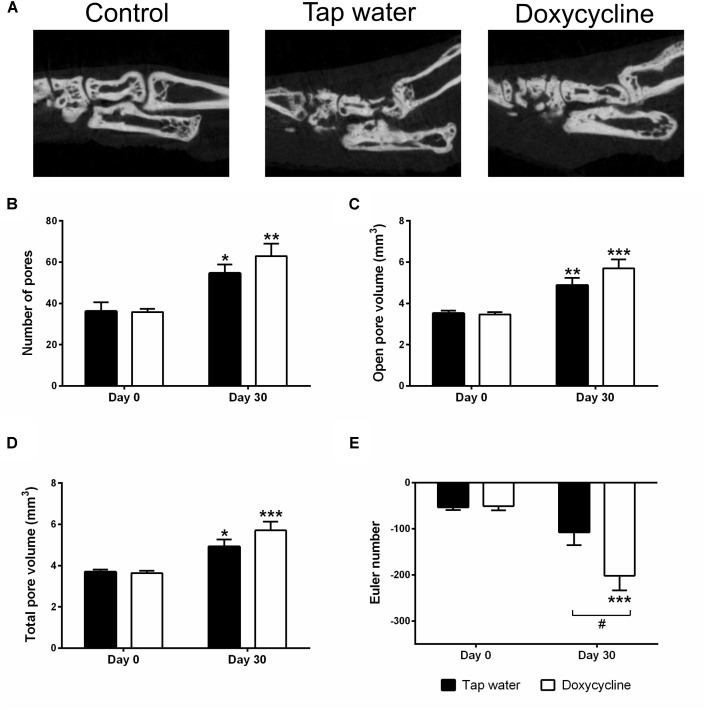
Doxycycline treatment does not affect arthritis-induced bone erosions around the ankle, but increased cancellous bone connectivity. **(A)** Representative sagittal CT slices of the ankle joints, **(B)** change of the number of pores, **(C)** volume of open pores, **(D)** the total volume of pores, and **(E)** the Euler number. Data are shown as means ± S.E.M. of *n* = 6–7 mice/group, ^∗^*p* < 0.05, ^∗∗^*p* < 0.01, ^∗∗∗^*p* < 0.001 vs. respective day 0 control, ^#^*p* < 0.05 vs. tap water consuming mice (two-way ANOVA followed by Sidak’s multiple comparison test).

**FIGURE 7:**
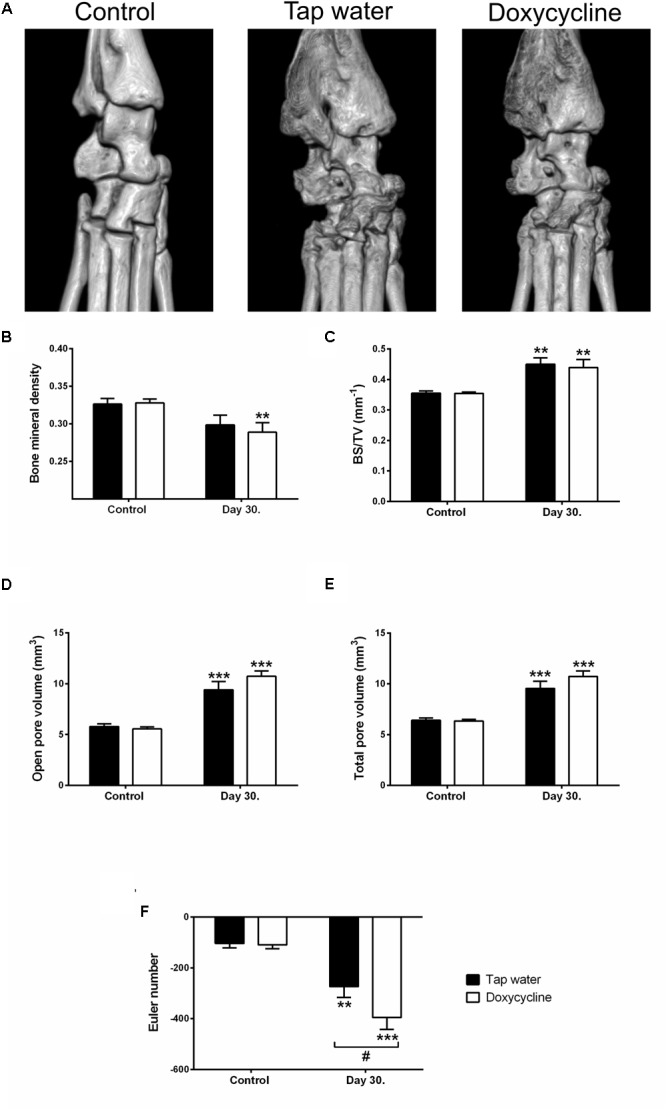
Doxycyline treatment decreases bone mineral density in the periarticular region of the distal tibia, while increasing trabecular connectivity, but has no effect on the formation of arthritic bone erosions. **(A)** Representative 3D micro-CT reconstructions of the right ankle joints, **(B)** bone mineral density, **(C)** bone surface density, **(D,E)** open and total pore volume, and **(F)** Euler number. Data are shown as means ± S.E.M. of *n* = 6–7 mice/group, ^∗∗^*p* < 0.01, ^∗∗∗^*p* < 0.001 vs. respective day 0 control, ^#^*p* < 0.05 vs. tap water consuming mice (two-way ANOVA followed by Sidak’s multiple comparison test).

The arthritis induced a marked irregularity of the ankle bones, narrowing of the tibio-tarsal joints, and widespread erosions, visually apparent on both CT slices and 3D reconstructions (Figure 6A, 7A). This was reflected by the quantitative analysis, which revealed that the number and volume of open and all pores increased significantly due to arthritis regardless of treatment with SDD in the ankle region (Figure 6B–D). Open and total pore volume also increased in the distal tibia (Figure 7D,E). Bone mineral density decreased in the periarticular region in SDD-treated mice, but not in their tap water consuming controls (Figure 7B). Bone surface density increased in both groups in the distal tibia, highlighting the marked osteophyte formation (Figure 7C). The Euler number, a measure of bone structural connectedness was significantly greater in SDD-treated animals in both regions (Figure 6E, 7F).

### Chronic Oral SDD Administration Results in Subantimicrobial Concentration in the Systemic Circulation

The plasma concentrations of doxycycline ranged from 0.036 to 0.151 μg/mL in the mice drinking the 0.5 mg/mL solution for 16 or 30 days (Table 1). The water consumption did not differ between respective tap water consuming and SDD-treated groups, but it was significantly lower in the arthritic groups as compared to non-arthritic controls as a sign of general sickness behavior (Supplementary Figure S1A). The original body weight of the mice was not significantly different (average 29 g), but after day 7 significant 15–25% weight loss was observed in both arthritic groups as compared to the non-arthritic ones. Furthermore, the weight loss of SDD drinking arthritic mice was significantly greater than the tap-water consuming arthritic ones during the period of days 7–10 (Supplementary Figure S1B).

### Increased MMP Activity in the Arthritic Ankle Joint Homogenates Was Not Influenced by Doxycycline

**FIGURE 8:**
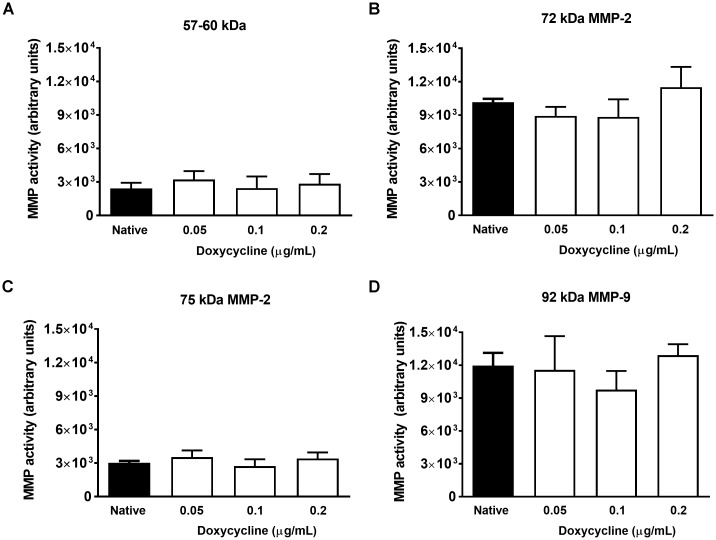
Doxycycline does not inhibit *in vitro* MMP activity in the arthritic joint homogenates. Changes of *in vitro* gelatinolytic activity of **(A)** 57–60 kDa, **(B)** 72 kDa, **(C)** 75 kDa, and **(D)** 92 kDa MMP isoforms in the arthritic and the non-arthritic control joint homogenates incubated with 0.05, 0.1, and 0.2 μg/mL doxycycline compared to the native control. Data are shown as means ± S.E.M. of *n* = 4 samples/group (one-way ANOVA followed by Bonferroni’s multiple comparison test).

All examined MMP isoform activities were enhanced in the doxycycline-free arthritic joint homogenates, but none of them changed after incubation with 0.05, 0.1, and 0.2 μg/mL doxycycline, the concentration range measured in the mouse plasma (Figure 8A–D). Among higher concentrations only the 200 μg/mL concentration which is remarkably above both the subantimicrobial concentration and the maximal concentration detected in the plasma, was able to significantly inhibit MMP-9, but not MMP-2 activity (Supplementary Figures S2A–D).

### Doxycycline Does Not Alter cPLA2 Activity and PGE2 Level in the Joints

Based on the observed worsening effect of SDD on arthritic bone structure deterioration and potential involvement of the prostanoid system in bone metabolism, we measured cPLA2 activity and PGE2 levels in the tibio-tarsal joint homogenates. On day 16 PLA2 activity, but not PGE2 concentration increased significantly in the arthritic joint homogenates of the tap water drinking group as compared to non-arthritic controls. In the late phase (day 30), neither cPLA2 activity, nor PGE2 concentrations were significantly elevated in the arthritic joints. SDD treatment did not influence these parameters at either timepoints (Supplementary Figure S3).

## Discussion

We showed here the increase of various MMP activities in the joints by *in vivo* fluorescence imaging together with *ex vivo* zymography, as well as investigated their functional significance using the broad spectrum MMP inhibitor SDD in the translational mouse model of RA. This is the first demonstration that SDD alters arthritic bone microarchitecture most probably independently of MMP inhibition.

Various MMP activities and their functional significance were examined in the K/BxN serum-transfer arthritis model, which is particularly appropriate to investigate the innate immune system-mediated effector phase and the chronic neuropathy-like pain component of arthritis (Korganow et al., 1999; Christianson et al., 2010). However, there are only two papers studying the role of specific MMPs in this model. The first paper shows that MMP-8 deficiency increases joint inflammation and bone erosion suggesting the protective role of MMP-8, while the second one demonstrates that the lack of MMP-13 decreases both clinical and histological severity of arthritis indicating the promoting function of MMP-13 in the disease (Garcia et al., 2010;Singh et al., 2013).

**Table 1:** Water consumption (mL/day/mouse; mean ± S.E.M.; *n* = 3–13 mice/group) and plasma doxycycline concentration (μg/mL; mean ± S.E.M.).

Group	Treatment	Water consumption (mL/day/mouse)	Plasma doxycycline concentration (μg/mL)
Control	Tap water	8.05 ± 0.33	n.a.
	Doxycycline	6.97 ± 0.26	0.066 ± 0.002
Arthritic	Tap water	4.09 ± 0.27	n.a.
	Doxycycline	3.79 ± 0.21	0.071 ± 0.002

*Consumption and plasma concentration data represent 3 mice in non-arthritic control, 12 mice in arthritic tap water-consumed group, 5 mice in non-arthritic control, 13 mice in arthritic doxycycline-treated group.*

In order to obtain a deeper and more complex insight about the activity and roles of various MMPs in this model, we applied integrative *in vivo* and *in vitro* methodology. Significantly increased MMP activity in the arthritic ankle joints was observed *in vivo* using a non-selective fluorescent imaging dye sensitive for MMP-2, -3, -7, -9, -12, and -13. In accordance with these findings, enhanced activities of a 57–60 kDa MMP, 75 kDa MMP-2, and 92 kDa MMP-9 isoforms were shown from the arthritic ankle joint homogenates by gelatin zymography *ex vivo*. The increased gelatinase activity at the band of 57–60 kDa was particularly surprising. There are three possible types of MMPs (proMMP-1, -3, and -13) that this can be related to, but the most likely candidates are proMMP-3 and proMMP-13. ProMMP-3 is secreted as a 57 kDa form, which can be glycosylated resulting in a 60 kDa protein (Wilhelm et al., 1987). Its presence is further suggested by the fact that it was also purified from human rheumatoid synovial fibroblasts (Okada et al., 1986). The molecular weight of the proMMP-13 is also 60–65 kDa. Its active form (MMP-13) is 50–55 kDa in size, but it is further cleaved into a final active form of 48 kDa (Freije et al., 1994; Knauper et al., 1996). MMP-13 is expressed by human chondrocytes (Blavier and Delaisse, 1995; Borden et al., 1996; Mitchell et al., 1996; Reboul et al., 1996), synovial membrane (Wernicke et al., 1996), synovial stroma (Lindy et al., 1997), and synovial fibroblasts (Westhoff et al., 1999). ProMMP-1 is also a potential candidate on the basis of the molecular weight, but its presence in the gel is unlikely, because MMP-1 cleaves gelatin about 40 times less effectively than MMP-13 (Knauper et al., 1996). It is worth mentioning that the mouse and human type MMP-1 are not the same, in mice the equivalent of human MMP-1 is MMP-1a, which is structurally similar, but differently expressed (Foley and Kuliopulos, 2014). Overall, we suggest that the 57–60 kDa gelatinase activity refers to proMMP-3 and/or proMMP-13 isoforms, although it could accurately be determined only by mass spectroscopy.

Several data support the relevance of these MMP isoforms in the pathogenesis of human and experimental RA. In RA patients, MMP-1 and MMP-3 in the serum, synovial tissue (ST) and fluid (SF), MMP-2 and MMP-7 in the SF, MMP-9 in the plasma and SF, MMP-12 and MMP-13 in the ST and SF have already been detected (Ahrens et al., 1996; Wernicke et al., 1996; Yoshihara et al., 2000; Moore et al., 2000; Tolboom et al., 2002; Green et al., 2003; Tchetverikov et al., 2003; Liu et al., 2004). In some cases, their distinct roles were also described in animal models. In a previous study, genetic ablation of MMP-2 resulted in an exacerbated level of collagen antibody-induced arthritis, while the lack of MMP-9 attenuated it compared to the wildtype mice indicating suppressive role of MMP-2 and pro-inflammatory role of MMP-9 in the process (Itoh et al., 2002). Interestingly, despite the convincing human data, the role of MMP-3 could not be confirmed in animal models, since disease severity was not altered in MMP-3-deficient mice in two antigen-induced arthritis models (Mudgett et al., 1998; van Meurs et al., 1999). In contrast, MMP-12-null mice showed more extensive articular inflammation and cartilage destruction associated with massive neutrophil infiltration in the collagen-induced arthritis (CIA) model suggesting the protective role of macrophage-driven MMP-12 in RA (Bellac et al., 2014). The deleterious role of MMP-13 was also proven with gene-deficient mice and a highly selective inhibitor. MMP-13 deficiency in the K/BxN serum-transfer arthritis model, as well as selective MMP-13 inhibitor treatment in severe combined immunodeficiency and CIA models resulted in significantly reduced joint inflammation and cartilage destruction (Jungel et al., 2010; Singh et al., 2013).

Since we have detected significantly increased K/BxN serum-induced MMP activation with both *in vivo* optical imaging and gelatin zymography, we investigated their functional relevance in the model with the widely used non-selective inhibitor SDD. Doxycycline belongs to the tetracycline family, whose MMP-inhibitory effect in subantimicrobial dose has been known for three decades (Greenwald et al., 1987). After decades of research its precise inhibitory mechanisms were also identified, it blocks primarily MMP activity by the chelation of the catalytic zinc and altering enzyme conformation, but also suppresses their gene expression and the proteolytic activation (Golub et al., 1998; Smith et al., 1999). Although 50% inhibitory concentrations of tetracyclines ranging from 5 to 500 μM had been shown to inhibit MMP-1, -2, -8, -9, and -13 *in vitro*, only MMP-13 activity was reduced by 5 μM doxycycline bioavailable in the tissues after oral administration (Smith et al., 1999). Due to its unique feature, i.e., the lack of antimicrobial actions, SDD became one of the main pathways of the development of synthetic MMP inhibitor drugs. Moreover, it was already approved by the U.S. Food and Drug Administration for the treatment of chronic periodontitis and acne rosacea (Golub et al., 2016). There are only few literature data when tetracyclines including doxycycline were administered in arthritis models and there are no papers for its chronic use. Oral tetracycline treatment suppressed metalloproteinase activity in arthritic tissue, but even very high doses failed to exhibit substantial anti-inflammatory efficacy (reduce joint swelling or paw diameter) in adjuvant-induced arthritis of the rat (Greenwald et al., 1992). Furthermore, acute oral pretreatment with doxycycline (3, 10, and 30 mg/kg) in an acute antigen (modified Bovine Serum Albumin)-induced arthritis model of the mouse dose-dependently inhibited mechanical hyperalgesia (Pinto et al., 2010). Clinical trials also proves its efficacy in arthritis (Greenwald, 2011; Golub et al., 2016), but primarily due to the lack of selectivity it did not become a widely used drug in RA therapy. However, it is a valuable tool to investigate the roles of arthritis-related MMPs in preclinical research.

In our study, despite the predictable daily water consumption of the mice and reaching subantimicrobial plasma concentration (Table 1), SDD did not inhibit either the *in vivo* MMP activity or the *ex vivo* activity of gelatinases derived from the arthritic ankle. The latter result has been confirmed by incubating the arthritic joint homogenates with three different doxycycline concentrations according to the plasma levels measured from the SDD-treated animals. When testing higher, antimicrobial concentrations of doxycycline much above the highest plasma concentration measured in our study, only the highest concentration was able to significantly inhibit MMP-9, but not MMP-2 activity. These results are in good accordance with similar data, which we obtained in lung and heart derived from mice chronically exposed to cigarette smoke, where SDD treatment did not alter MMP activity *ex vivo*. However, *in vitro* treatment only with the highest plasma concentration measured from SDD-treated animals (0.24 μg/mL) was able to significantly decrease MMP-9, but not MMP-2 activity in the lung. Meanwhile, in the heart, none of the doxycycline concentrations decreased MMP-2 activity similarly to the joints (unpublished data). Although plasma doxycycline concentrations have been reliably determined, one limitation of this study is that we could not measure the tissue concentration of doxycycline in the joint homogenates due to technical difficulties. In accordance with MMP activity results none of the K/BxN serum-induced mechanical hyperalgesia, clinical signs, joint function impairment, neutrophil MPO activity and vascular hyperpermeability were altered by the SDD treatment.

Although characteristic functional symptoms accompanied by significantly increased MMP activity in the joints cannot be inhibited by SDD treatment in our chronic arthritis model, it has profound effects on inflammatory homeostatic imbalance of the bones. In our study significant decrease of bone mineral density, which is also a hallmark clinical feature of human RA, was only demonstrated in the region of the distal tibia of SDD-treated mice. Open porosity representing bone erosions increased in K/BxN serum-transfer arthritis model similarly in both SDD and tap water consuming animals. Bone surface density only increased significantly in the distal tibia, which can be explained by the osteophyte-formation in this region. Arthritis was accompanied by an increased trabecular connectivity in both distal tibia and ankle that is likely to reflect bone neoformation due to inflammation. SDD treatment further significantly enhanced this parameter in both regions of arthritic mice as compared to their tap water consuming controls suggesting that SDD treatment results in detrimental overall effect as shown by aggravated periarticular bone resorption and reactive bone remodeling.

Previous studies have shown no effects of SDD on bone architecture under healthy conditions (Fowlkes et al., 2015), but profoundly improved bone homeostasis in ovariectomy-induced osteopenia by inhibiting not only MMPs, but other collagenases involved in bone resorption (Pytlik et al., 2004). Furthermore, doxycycline can shift the bone homeostasis toward the osteoblastic pathway by directly inhibiting osteoclasts and facilitating their apoptosis via inhibiting the Dickkopf-related protein 1 (Dkk-1) pathway (Gomes et al., 2017). Since Dkk-1 overexpressing mice display an osteopenic phenotype, and anti-Dkk-1 antibody treatment prevents bone loss in experimental OA, interference with these signaling pathways is likely to be involved in the increased trabecular connectivity observed in SDD-treated mice in our experiment (Funck-Brentano et al., 2014). Another potential mechanism of SDD on arthritic bone structure deterioration may be PLA2 inhibition and the decreased level of PGE2, which plays an important role in bone metabolism (Lisowska et al., 2018). It is well-known that tetracyclines inhibit PLA2 activity and can theoretically decrease PGE2 level in bones (Pruzanski et al., 1992, 1998), but our present results did not confirm this concept. PLA2 activity, but not PGE2 concentration increased significantly in the arthritic joint homogenates of the tap water drinking group on day 16, when stable inflammatory symptoms such edema and hyperemia were present as compared to non-arthritic controls. In the late phase (day 30), when the inflammation was over and the arthritic bone structure deterioration was observed, neither PLA2 activity, nor PGE2 levels elevated. However, SDD administration did not influence these parameters at either timepoint.

## Conclusion

In conclusion, K/BxN serum-transfer arthritis model is characterized by significantly increased MMP levels, but the widely used non-selective MMP inhibitor SDD is not likely to inhibit MMP activity in the joints. SDD clearly worsens the chronic arthritis-induced bone microarchitectural alterations in a complex manner by simultaneously decreasing mineralization and increasing the trabecular connectivity.

## Data Availability

The raw data supporting the conclusions of this manuscript will be made available by the authors, without undue reservation, to any qualified researcher.

## Author Contributions

ÁH performed the evaluation of mechanical hyperalgesia, arthritis severity and joint function impairment, the preparation of doxycycline-treated drinking water, the measurement of water consumption, and wrote the manuscript. BB and ÁH carried out the *in vivo* bioluminescence and fluorescence imaging and assisted in data analysis, in study design and in writing the manuscript. TK and BB performed the micro-CT imaging, analyzed the data, and drafted the paper. KC helped to prepare the doxycycline-treated drinking water, participated in study design, and revised the manuscript. IK and AF performed the measurement of plasma concentration of doxycycline and drafted the manuscript. TS, ÉK, and PB carried out the gelatin zymography, PLA2 activity and PGE2 concentration measurements, helped to design the study, and to write the manuscript. AM provided K/BxN and BxN sera and revised the paper. PF and ZH designed the experiments, assisted in data analysis, and in writing the manuscript. All authors read and approved the final manuscript.

## Conflict of Interest Statement

The authors declare that the research was conducted in the absence of any commercial or financial relationships that could be construed as a potential conflict of interest.

## Supplementary Material

FIGURE S1 **(A)** Water consumption (mL/day/mouse) and **(B)** percentage change of body weight (%). Data are shown as means ± S.E.M. of *n* = 3–13 mice/group, ^∗^*p* < 0.05 vs. respective non-arhritic control mice, ^###^*p* < 0.001 vs. tap water consuming arthritic mice (two-way ANOVA followed by Bonferroni’s multiple comparison test).

FIGURE S2 Doxycycline in high concentration (200 μg/mL), but not in lower ones inhibits *in vitro* MMP-9 activity in the arthritic joint homogenates. Changes of *in vitro* gelatinolytic activity of **(A)** 57–60 kDa, **(B)** 72 kDa, **(C)** 75 kDa, and **(D)** 92 kDa MMP isoforms in the arthritic and the non-arthritic control joint homogenates incubated with 0.2, 2, 20, and 200 μg/mL doxycycline compared to the native control. Data are shown as means ± S.E.M. of *n* = 4 samples/group, ^∗^*p* < 0.05 vs. native control (one-way ANOVA followed by Bonferroni’s multiple comparison test).

FIGURE S3 **(A)** Cytosolic phospholipase A2 activity and **(B)** prostaglandin E2 levels of tibio-tarsal joint homogenates. Data are shown as mean ± S.E.M. of *n* = 3–7/group, ^∗∗^*p* < 0.01 vs. respective non-arthritic controls (one-way ANOVA followed by Bonferroni’s multiple comparison test).

## Abbreviations

ANOVA: analysis of variance
CIA: collagen-induced arthritis
CT: computed tomography
Dkk-1: Dickkopf-related protein 1
I.p.: intraperitoneal
LC-MS: liquid chromatography-mass spectrometry
MMP: matrix metalloproteinase
MPO: myeloperoxidase
MT: membrane-type
OA: osteoarthritis
PGE2: prostaglandin E2
PLA2: phospholipase A2
RA: rheumatoid arthritis
ROI: region of interests
SDD: subantimicrobial dose doxycycline
SF: synovial fluid
ST: synovial tissue.
