# Supplementary figures and images for: Subantimicrobial Dose Doxycycline Worsens Chronic Arthritis-Induced Bone Microarchitectural Alterations in a Mouse Model: Role of Matrix Metalloproteinases?
Source: Front Pharmacol. 2019 Mar 20;10:233. doi: 10.3389/fphar.2019.00233 (PMC6435543; doi:10.3389/fphar.2019.00233)

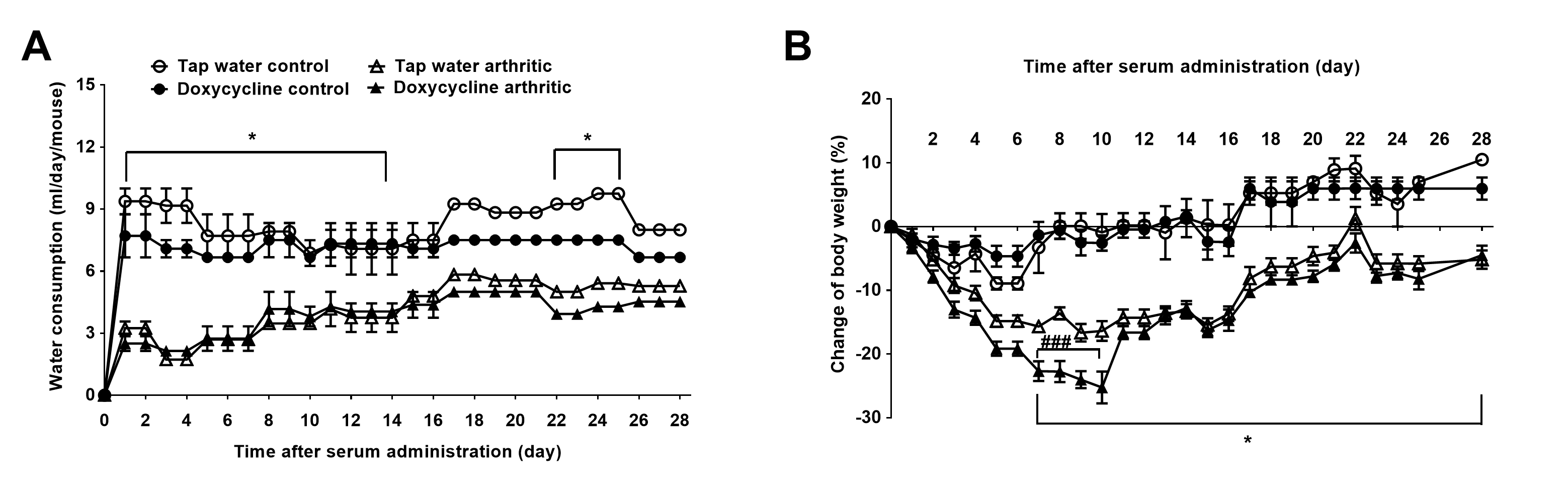

Supplement: FIGURE S1 — (A) Water consumption (mL/day/mouse) and (B) percentage change of body weight (%). Data are shown as means ± S.E.M. of n = 3–13 mice/group, ∗p < 0.05 vs. respective non-arhritic control mice, ###p < 0.001 vs. tap water consuming arthritic mice (two-way ANOVA followed by Bonferroni’s multiple comparison test). [file Image_1.TIF]

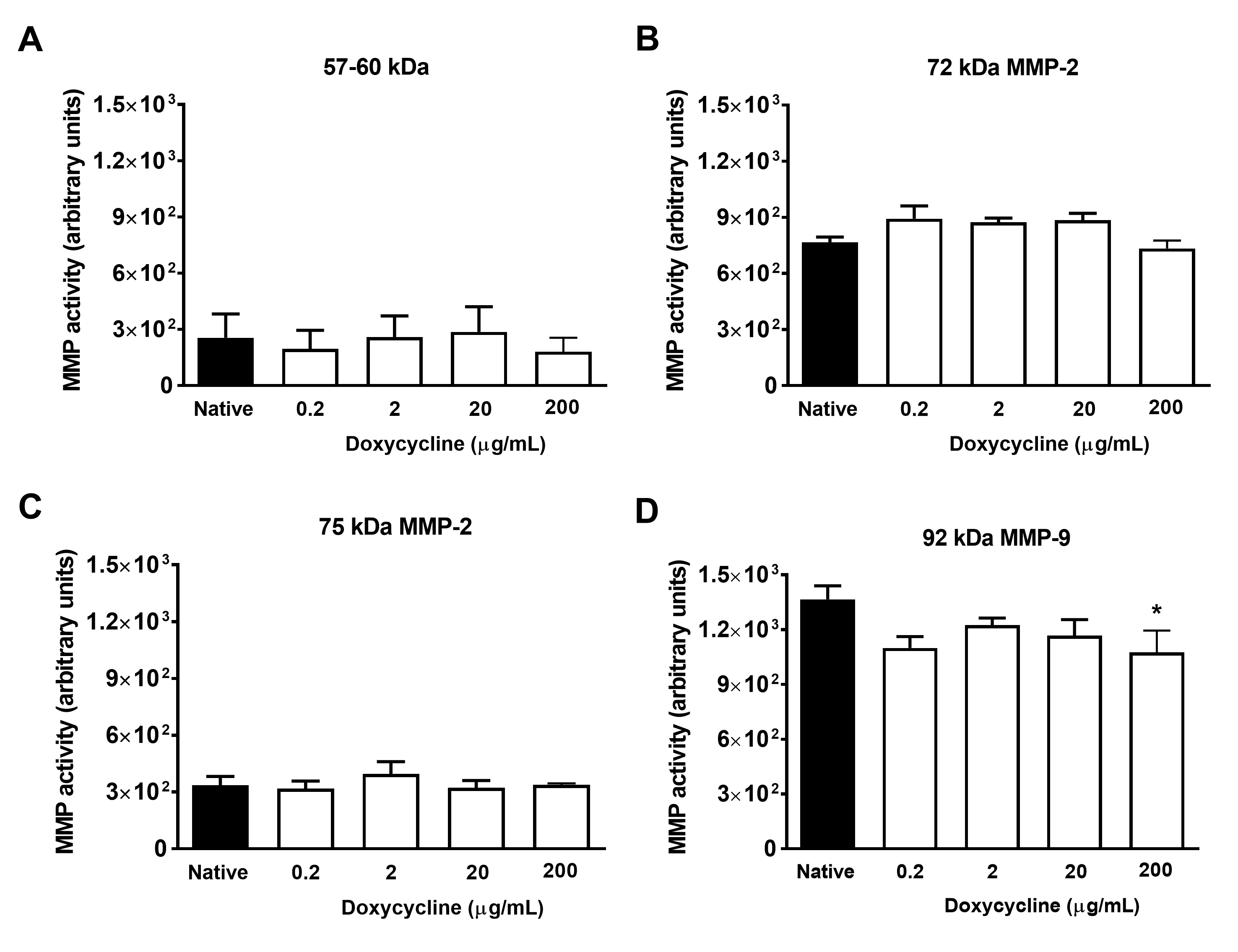

Supplement: FIGURE S2 — Doxycycline in high concentration (200 μg/mL), but not in lower ones inhibits in vitro MMP-9 activity in the arthritic joint homogenates. Changes of in vitro gelatinolytic activity of (A) 57–60 kDa, (B) 72 kDa, (C) 75 kDa, and (D) 92 kDa MMP isoforms in the arthritic and the non-arthritic control joint homogenates incubated with 0.2, 2, 20, and 200 μg/mL doxycycline compared to the native control. Data are shown as means ± S.E.M. of n = 4 samples/group, ∗p < 0.05 vs. native control (one-way ANOVA followed by Bonferroni’s multiple comparison test). [file Image_2.TIF]

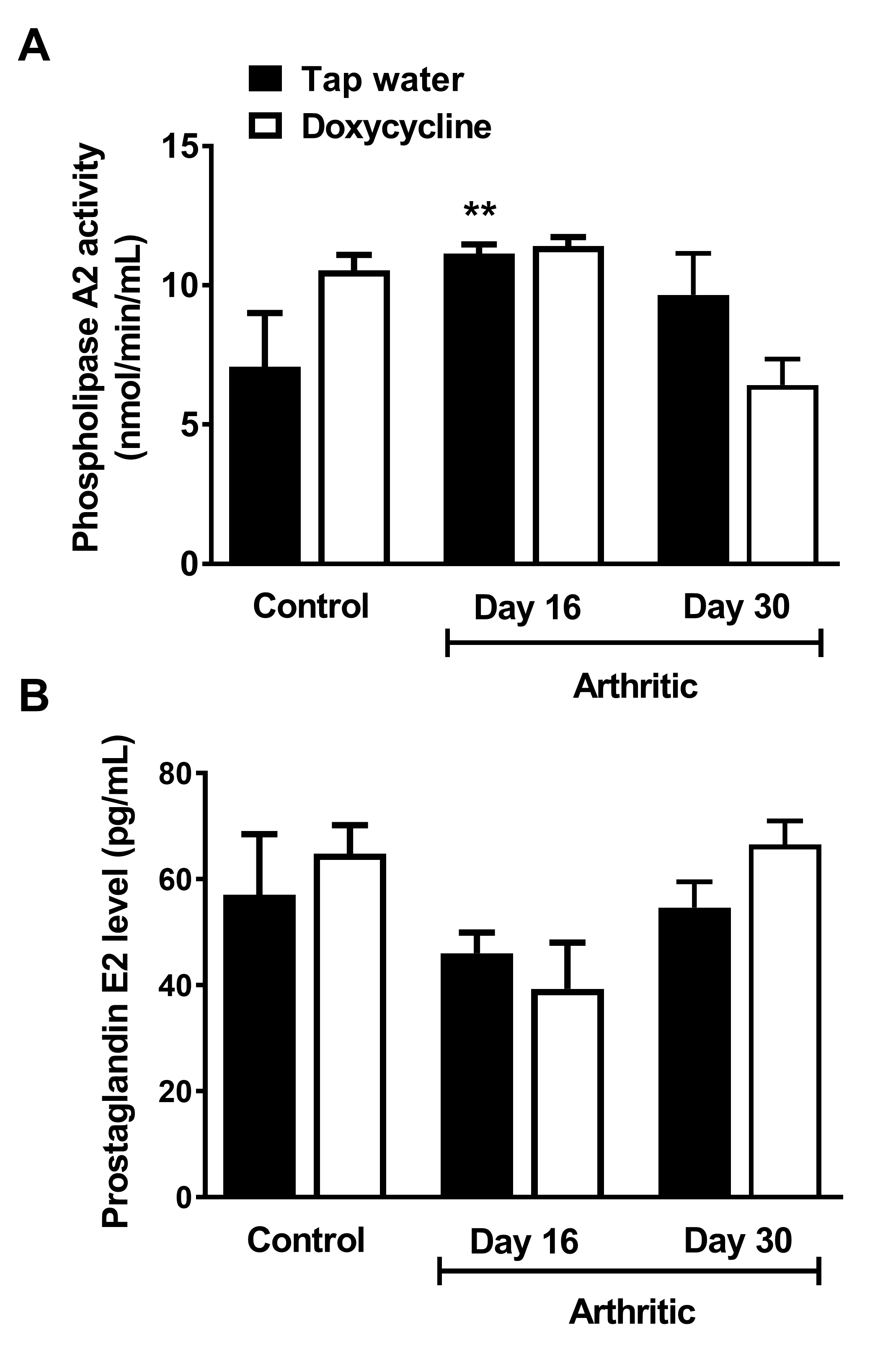

Supplement: FIGURE S3 — (A) Cytosolic phospholipase A2 activity and (B) prostaglandin E2 levels of tibio-tarsal joint homogenates. Data are shown as mean ± S.E.M. of n = 3–7/group, ∗∗p < 0.01 vs. respective non-arthritic controls (one-way ANOVA followed by Bonferroni’s multiple comparison test). [file Image_3.TIF]
